# Supplementary material for: Near-complete genomes for nine haplochromine cichlid fishes reveal a novel centromeric satellite structure organised around a pair of inverted elements
Source: Chromosome Res. 2026 Oct 9;34(1):28. doi: 10.1007/s10577-026-09819-0 (PMC13652916; doi:10.1007/s10577-026-09819-0)

Near-complete genomes for nine haplochromine cichlid fishes reveal a novel centromeric satellite structure organised around a pair of inverted elements.

Pío Sierra, Chexi Zhou, Bettina Fischer, Sing Wei Lim, Moritz Blumer, Maxon Ngochera, Richard Durbin.

Supplementary figures.-

Supp. Figs. 8 to 29.- Self dotplots of 3 MB centered on the centromere of each chromosome for each of the nine species.

Supp. Fig. 30.- Correlation between centromere size and presence or absence of centroids, in ungapped and gapped centromeres.

Supp. Fig. 31.- Satellite sequences from one chromosome aligned keeping their relative order in the centromere. The satellites belong, top down, to a region 5' of external satellites, 5' centroid, 5' internal satellites, 3' internal satellites, 3' centroid, 3' external satellites. The vertical arrows indicate examples of nucleotide variants which appear to be particular of just one of the segments.

Supp. Fig. 8.- Centromeres of chr 1

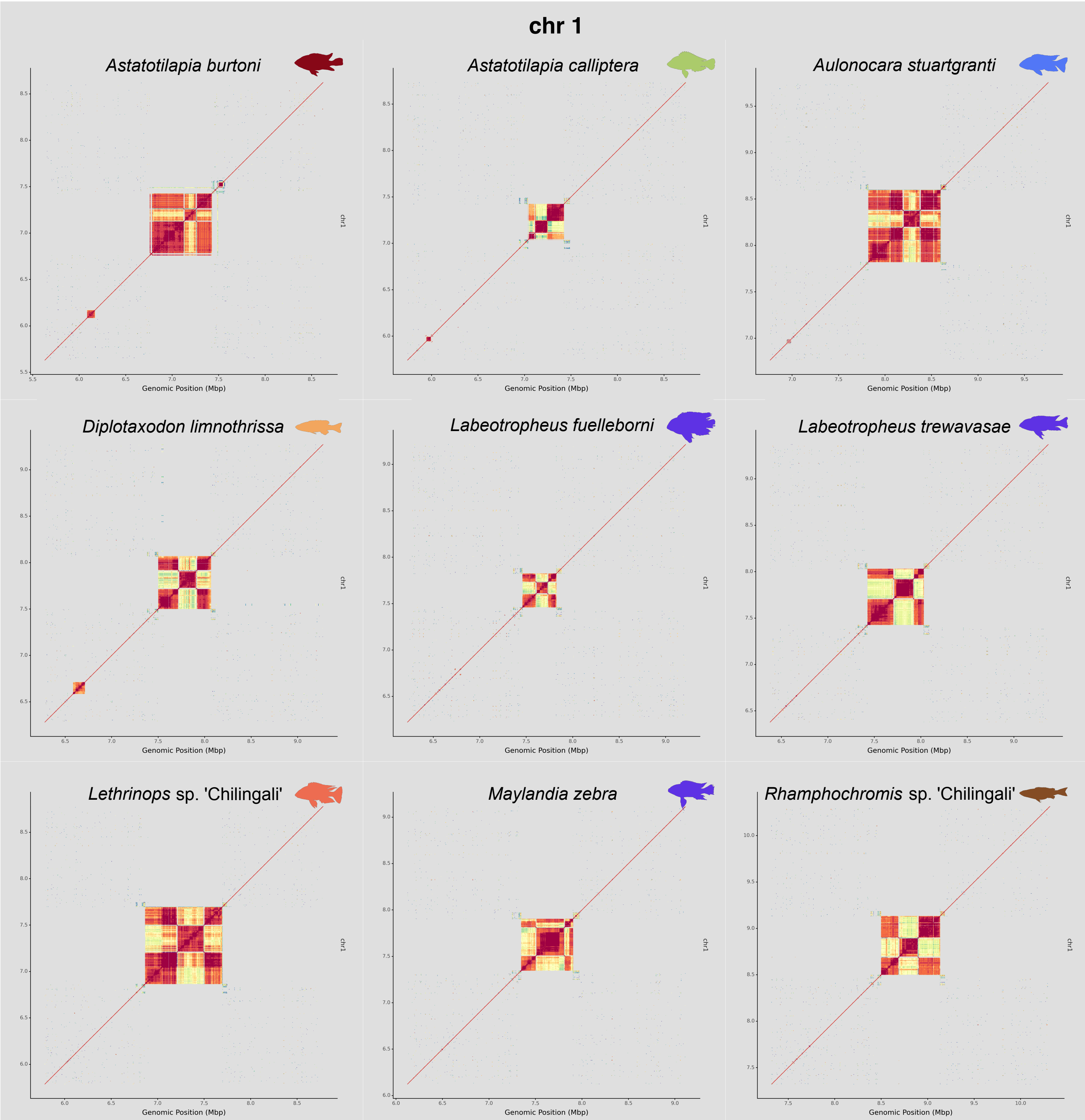

Supp. Fig. 9.- Centromeres of chr 2

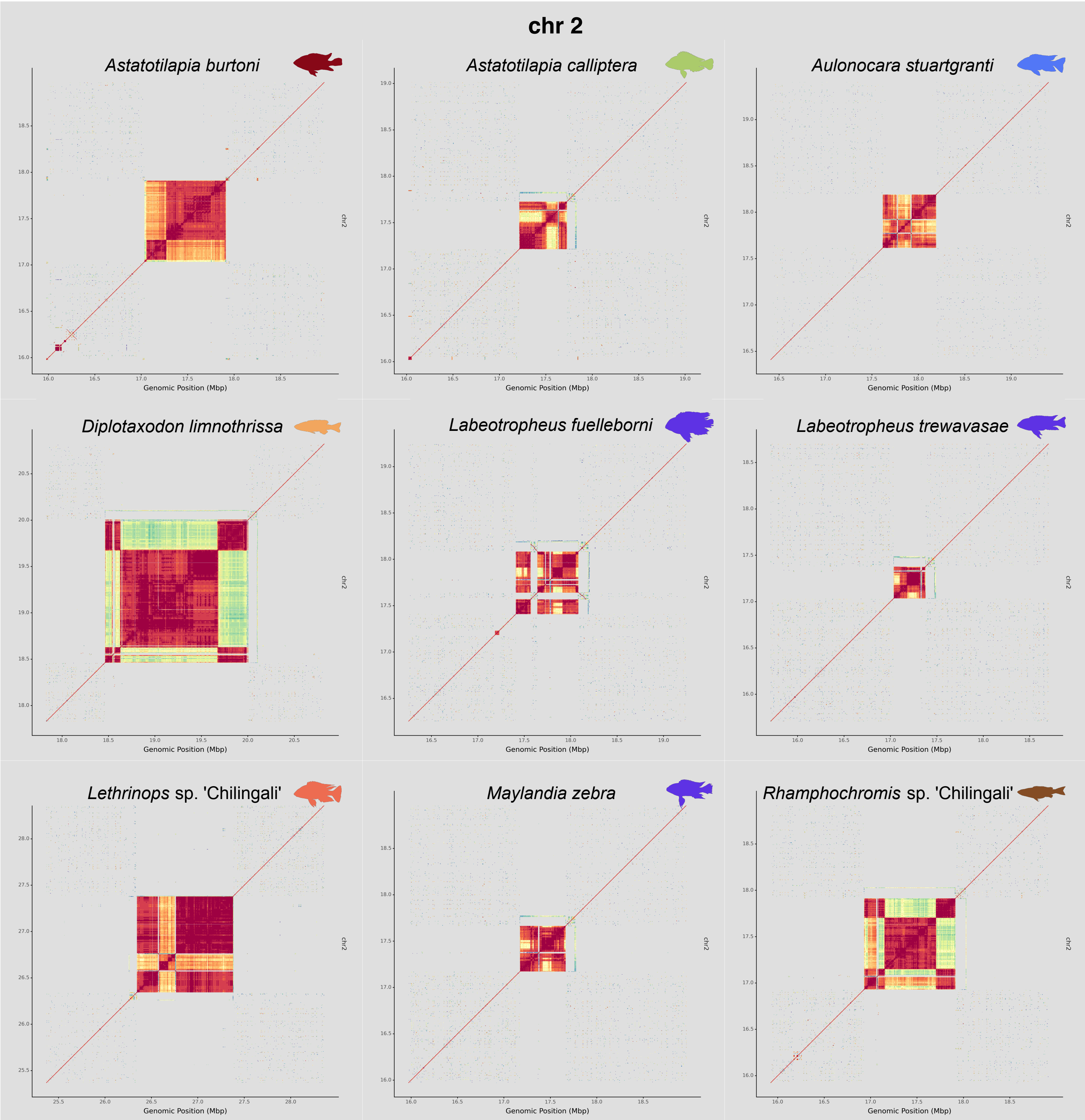

Supp. Fig. 10.- Centromeres of chr 3

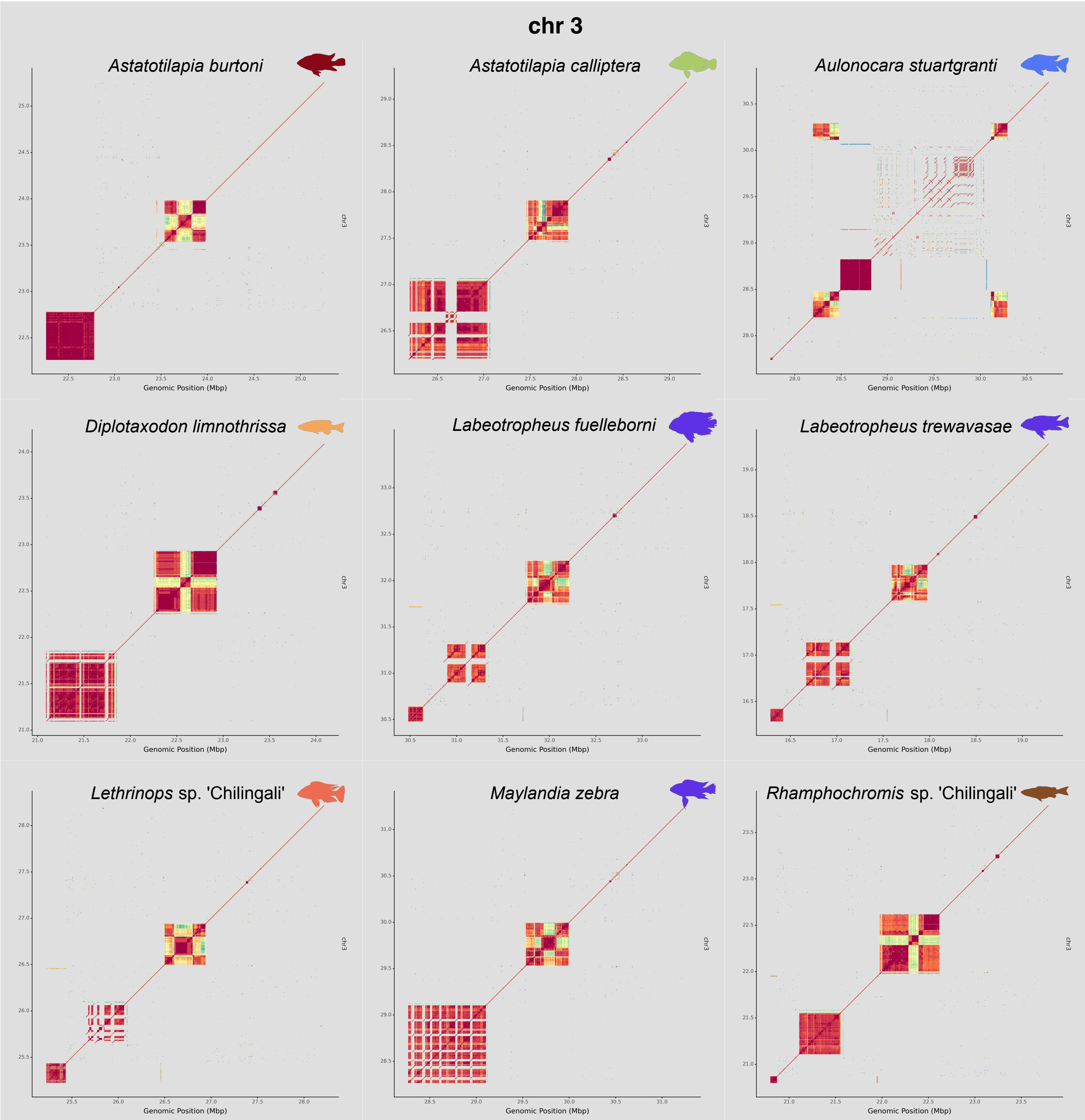

Supp. Fig. 11.- Centromeres of chr 4

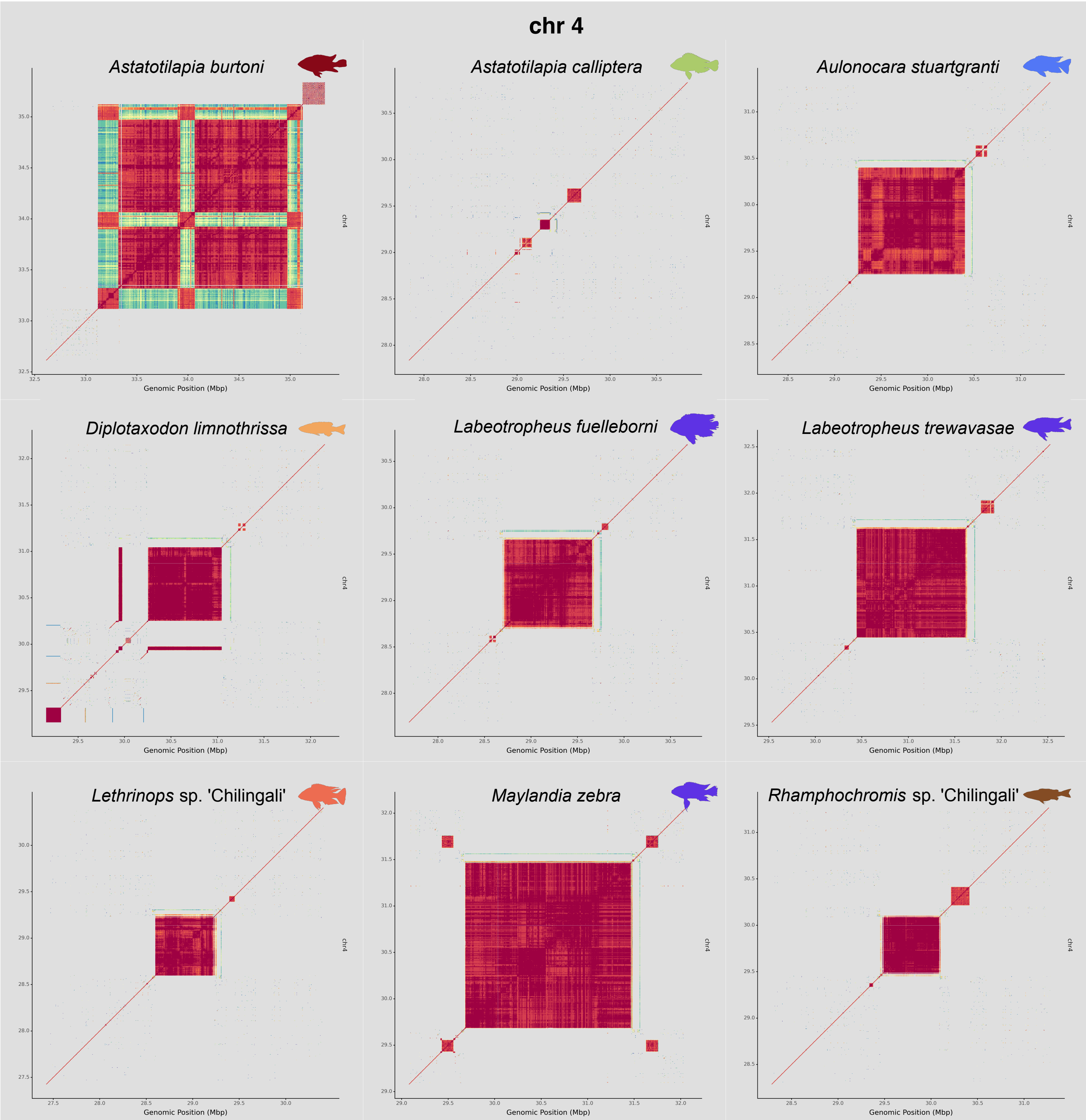

Supp. Fig. 12.- Centromeres of chr 5

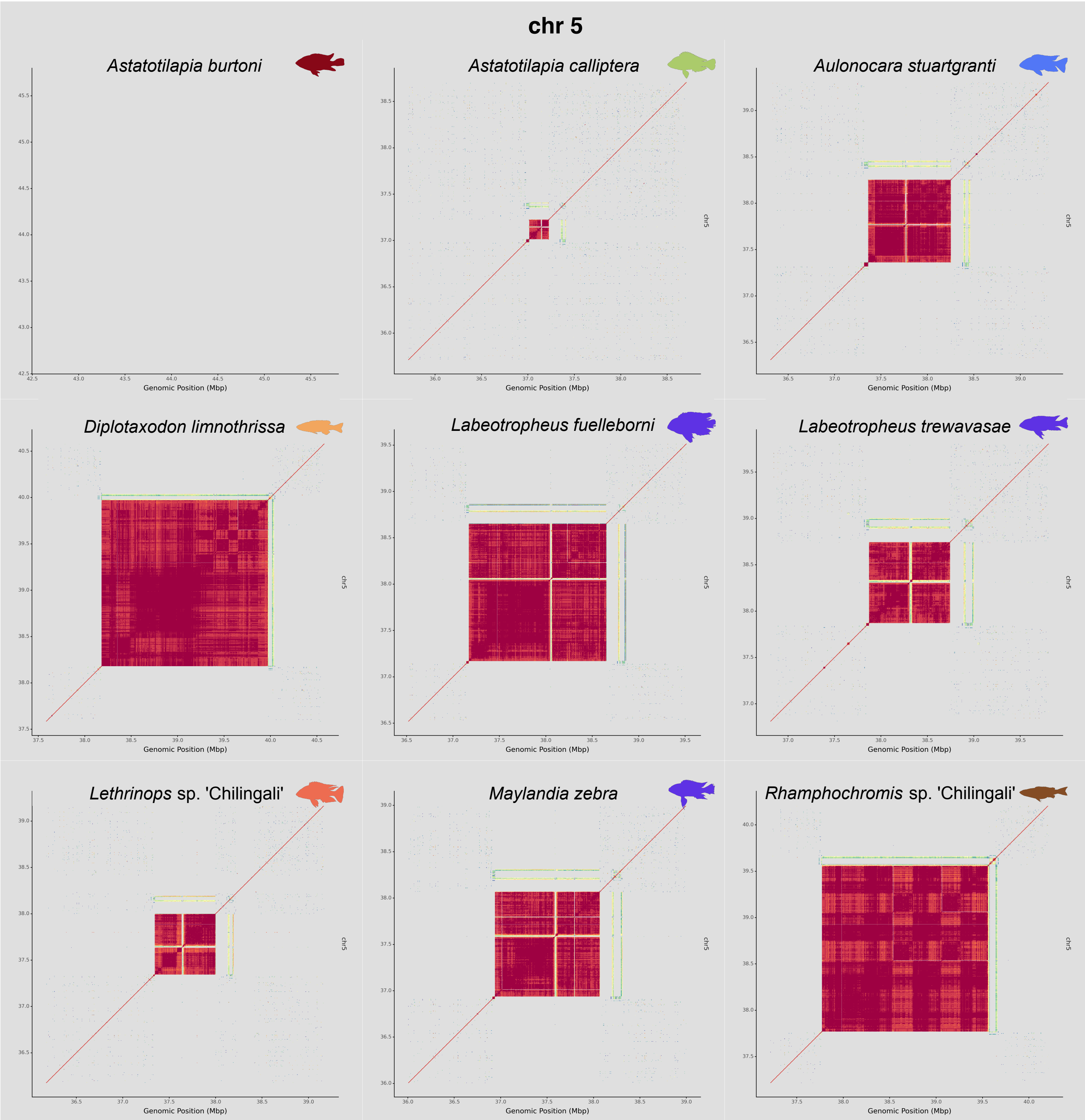

Supp. Fig. 13.- Centromeres of chr 6

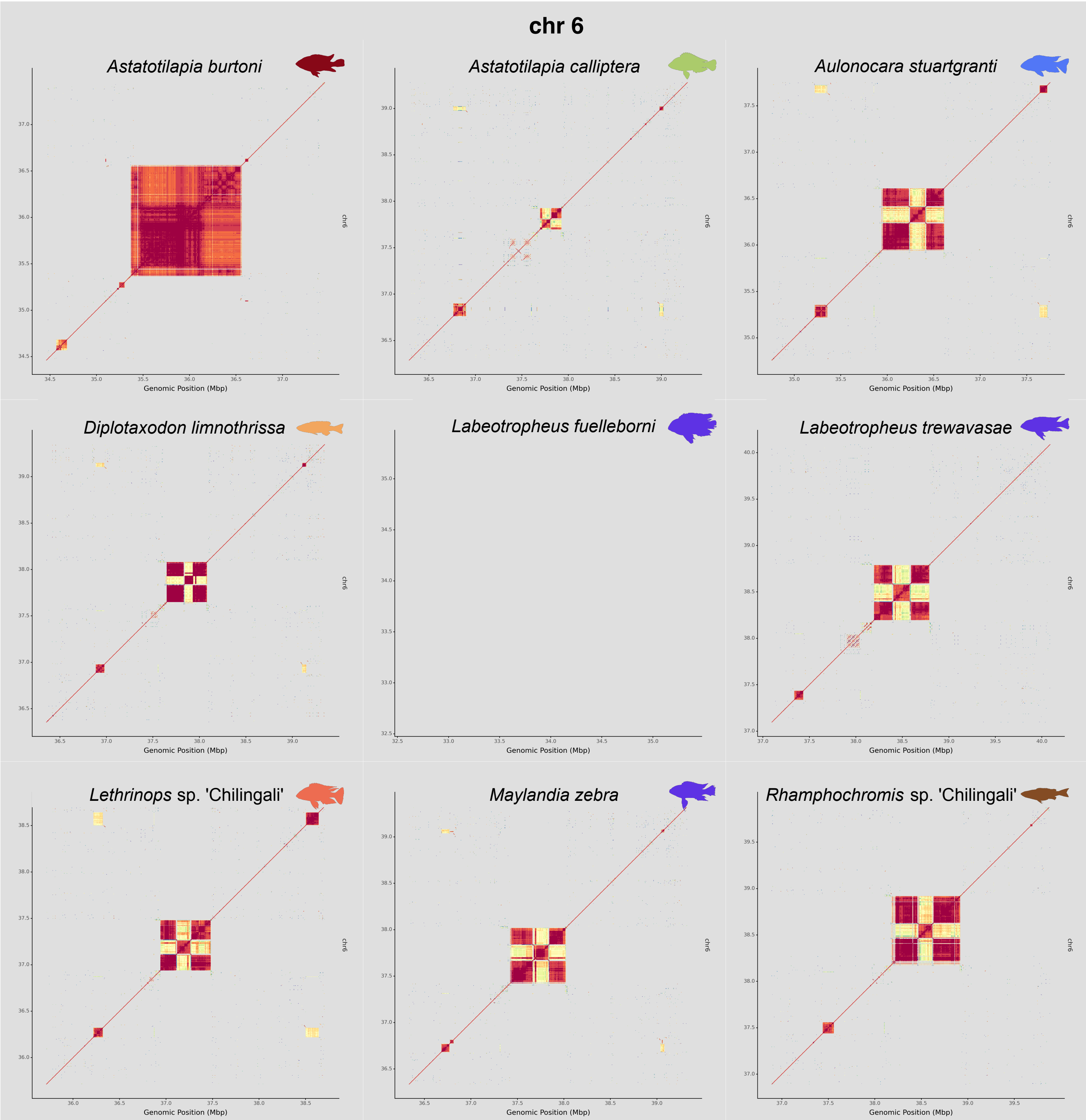

Supp. Fig. 14.- Centromeres of chr 7

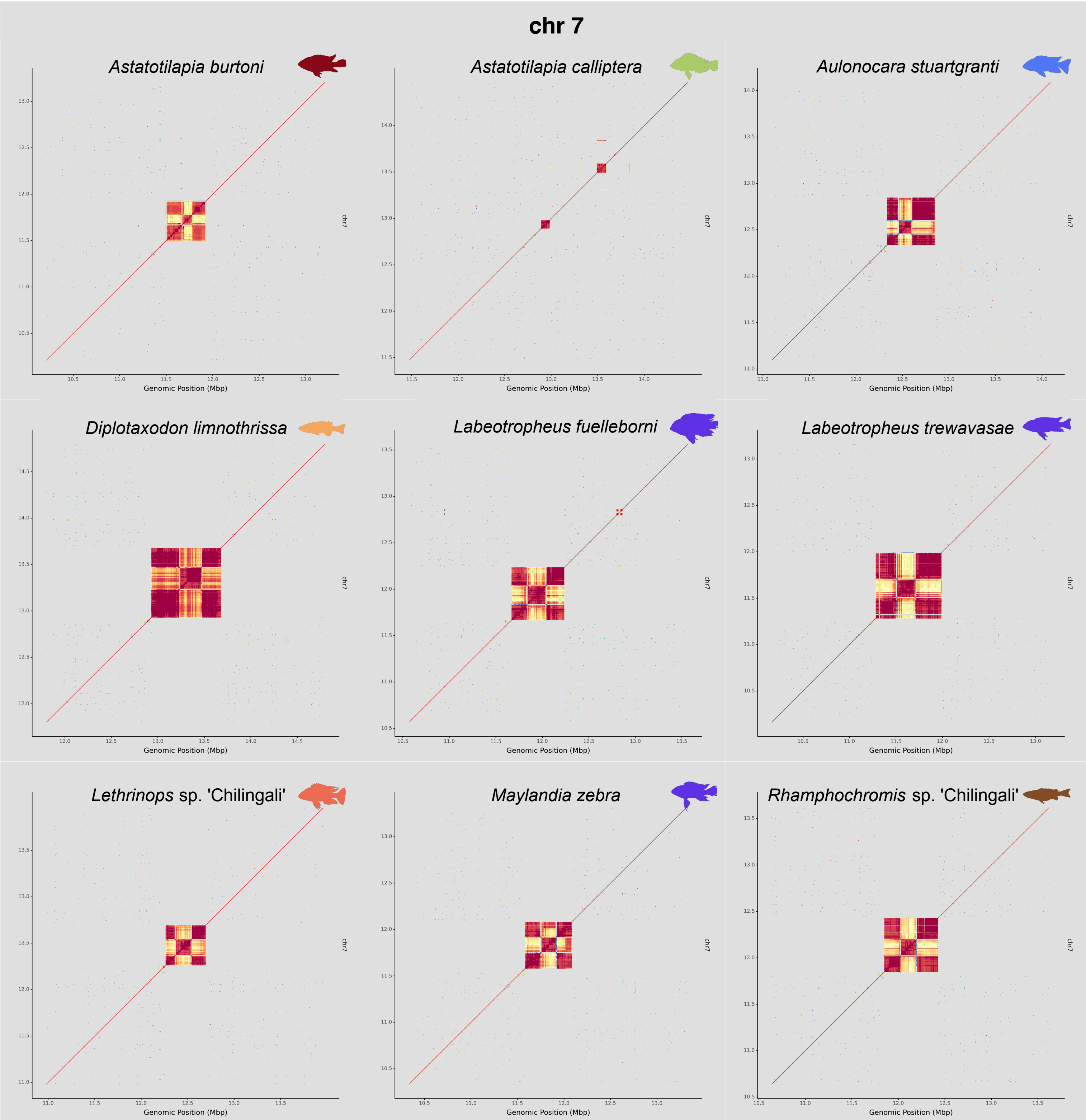

Supp. Fig. 15.- Centromeres of chr 8

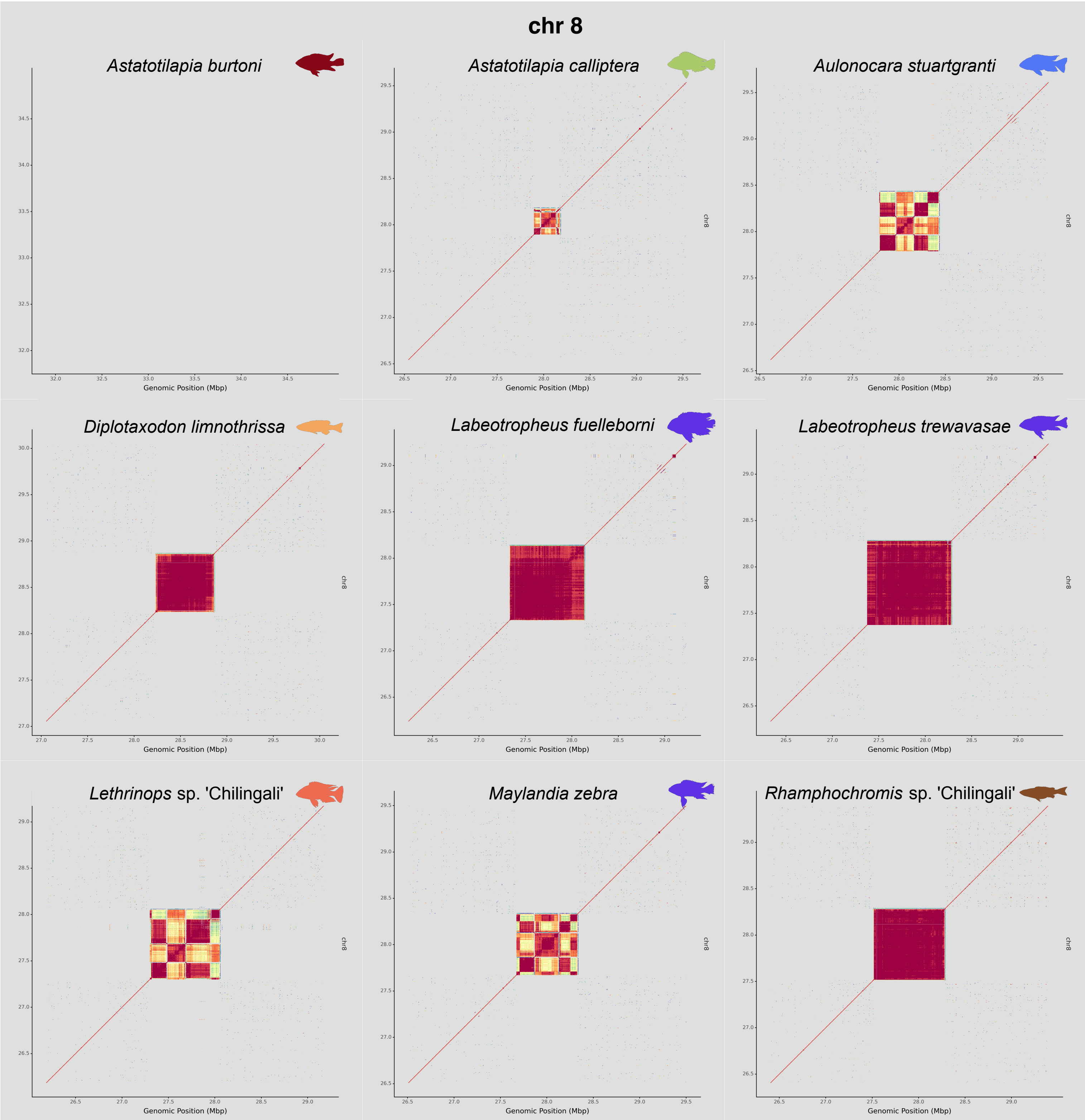

Supp. Fig. 16.- Centromeres of chr 9

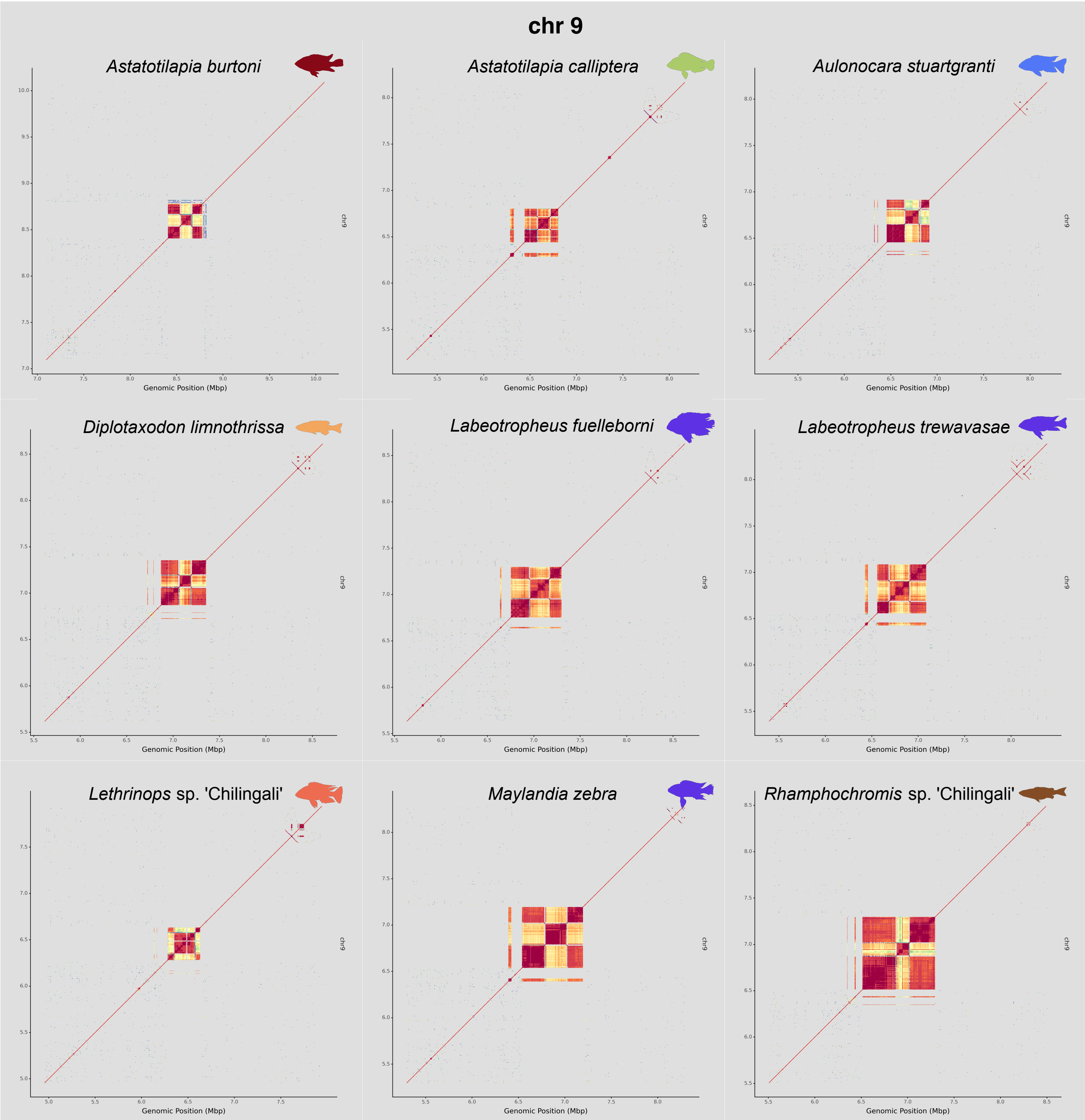

Supp. Fig. 17.- Centromeres of chr 10

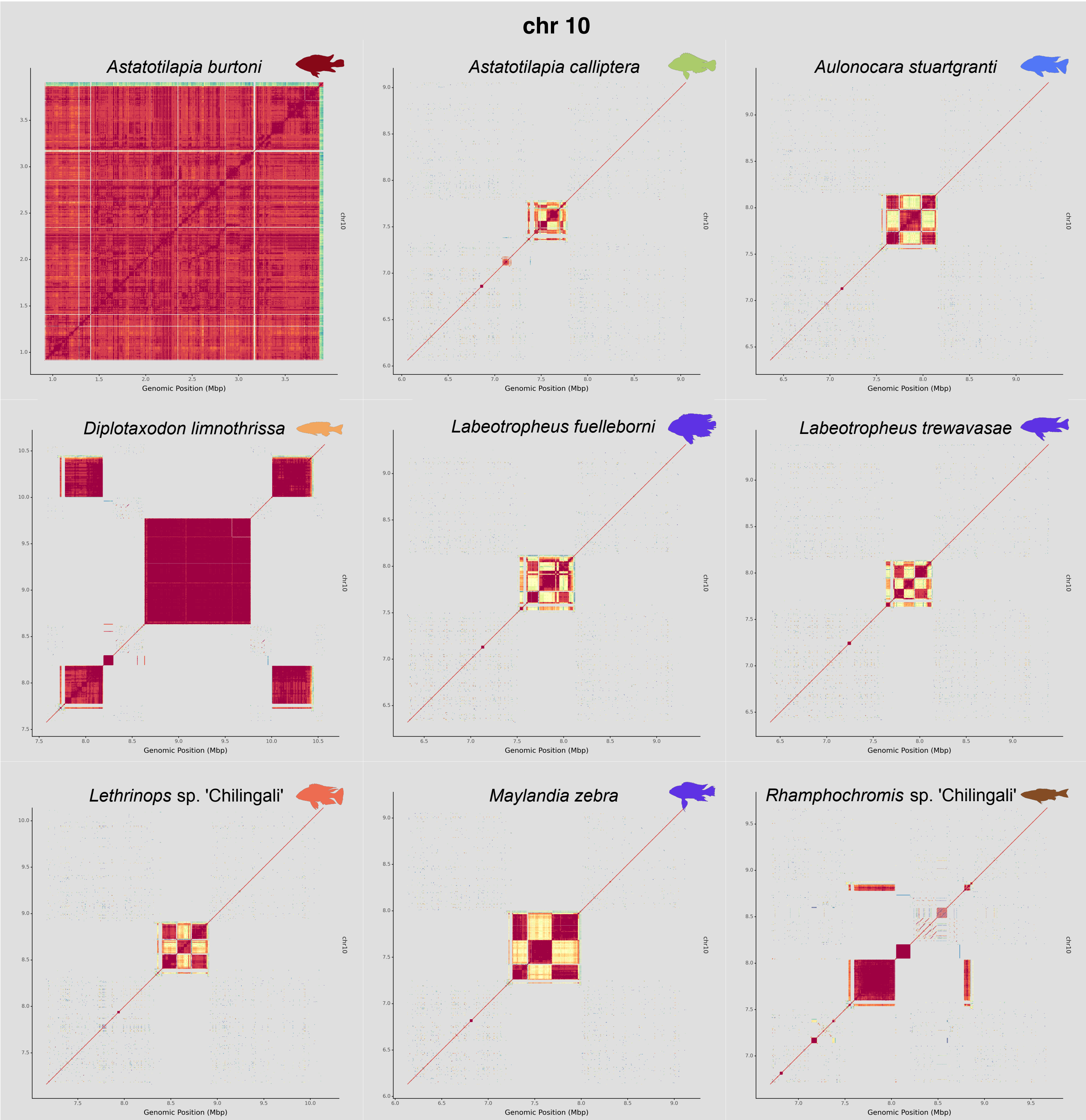

Supp. Fig. 18.- Centromeres of chr 11

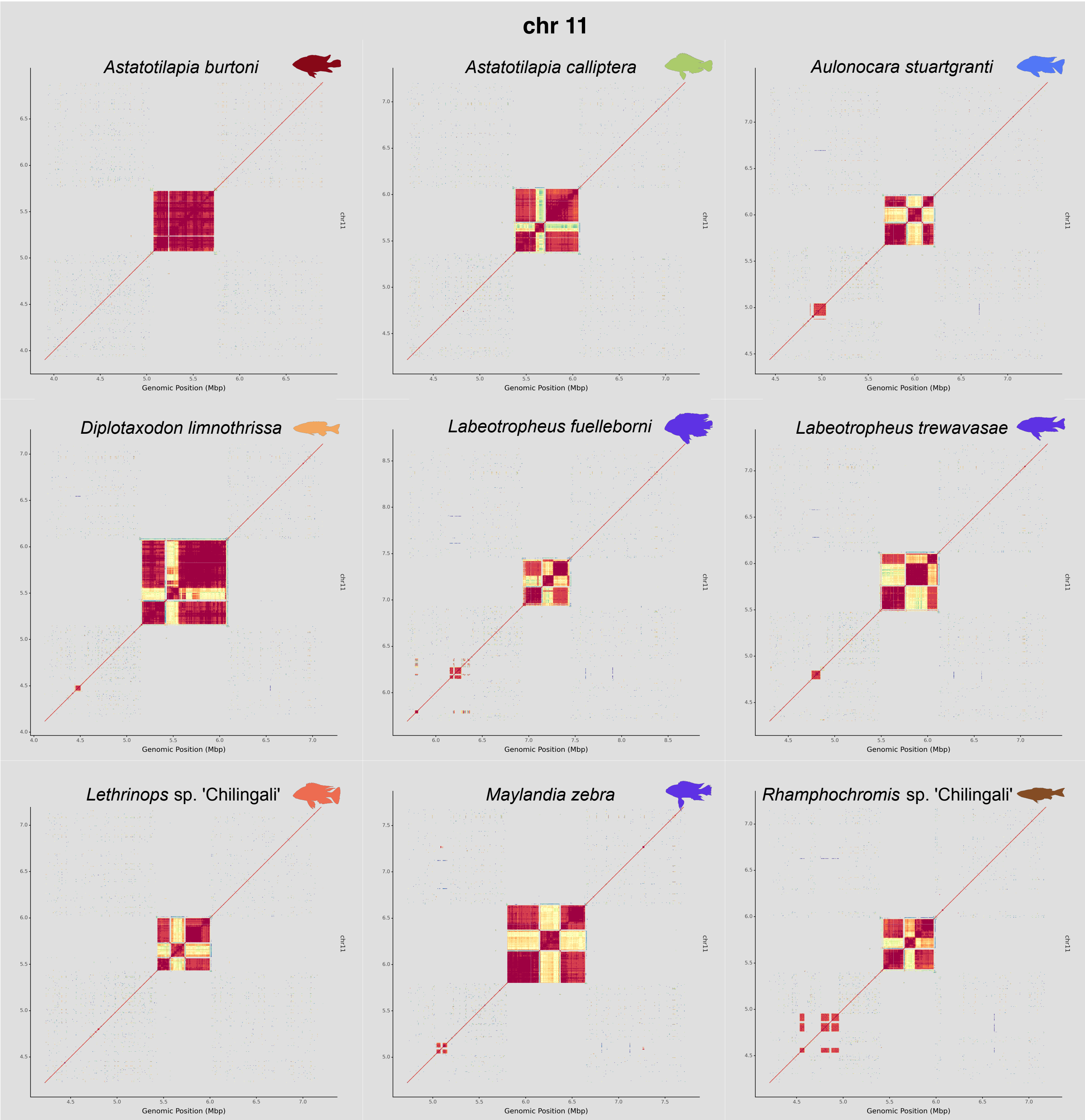

Supp. Fig. 19.- Centromeres of chr 12

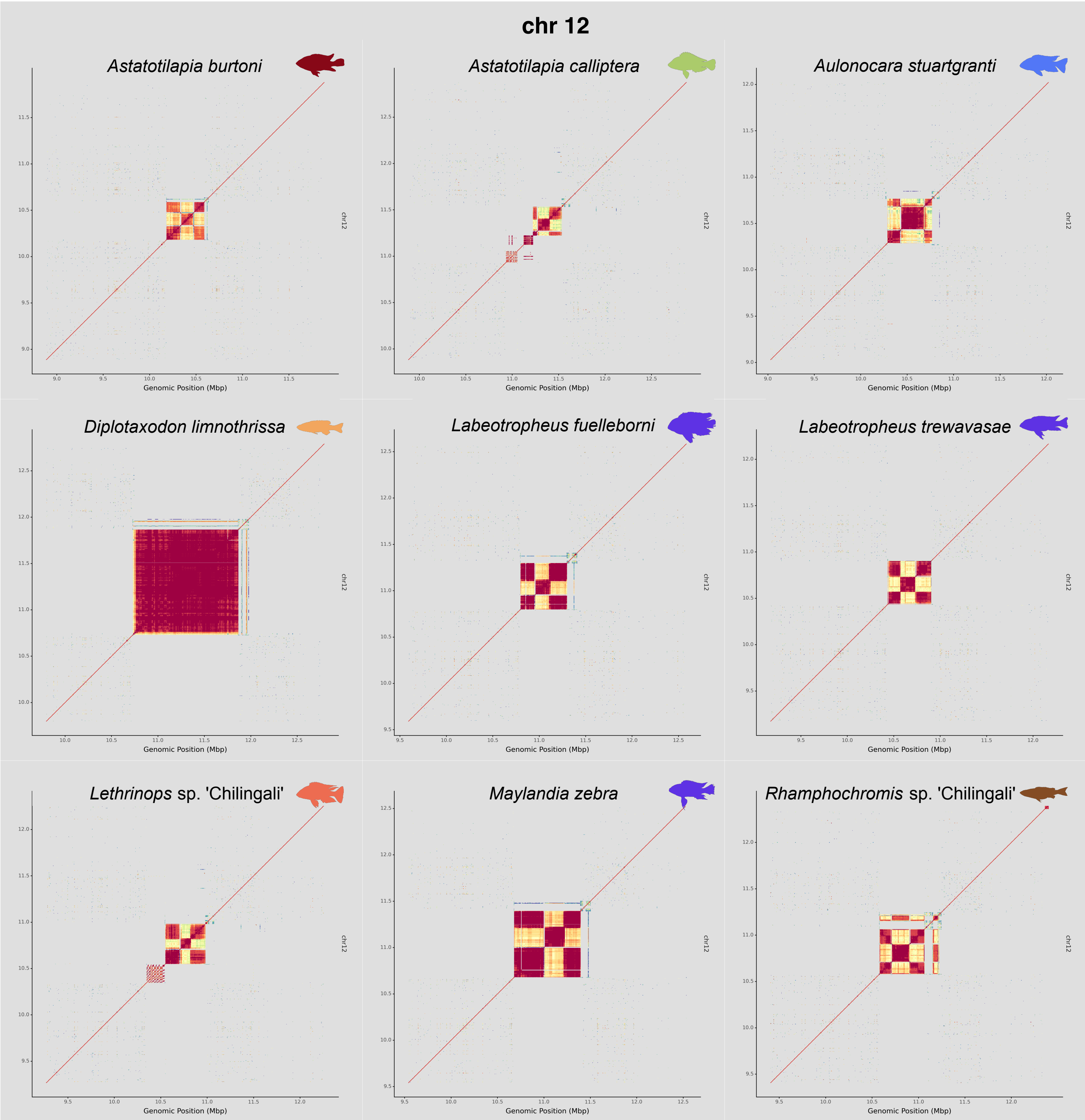

Supp. Fig. 20.- Centromeres of chr 13

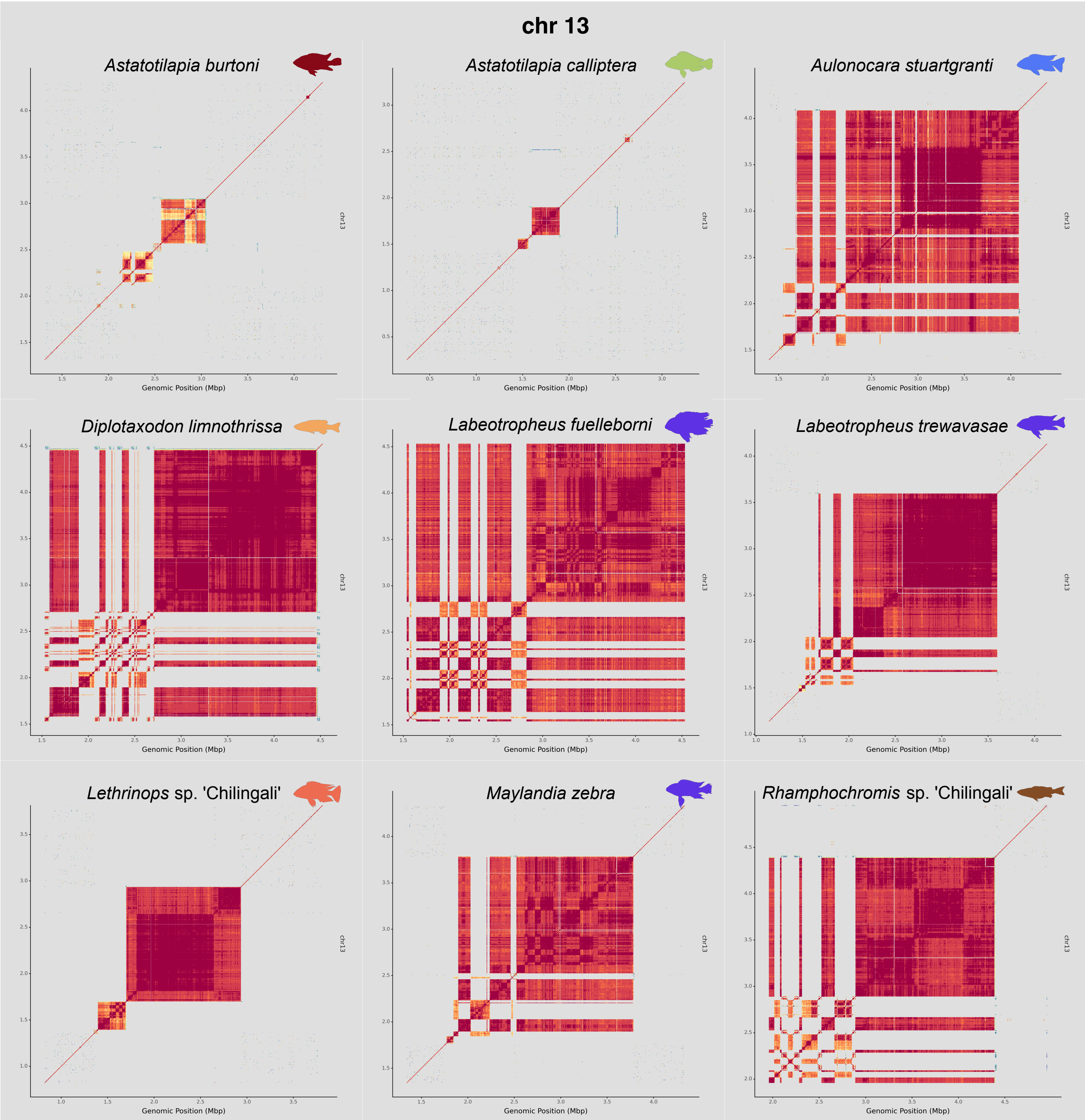

Supp. Fig. 21.- Centromeres of chr 14

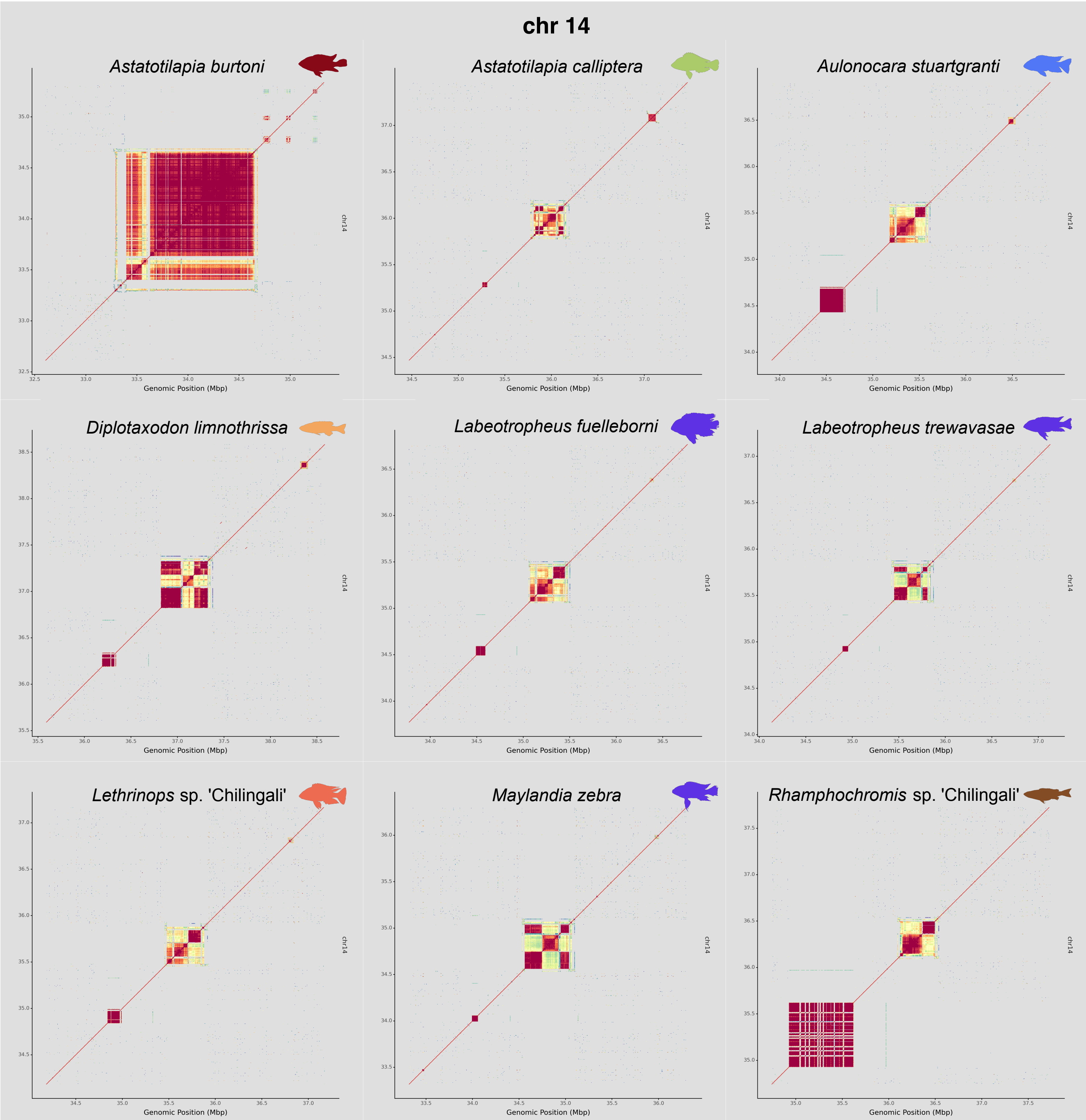

Supp. Fig. 22.- Centromeres of chr 15

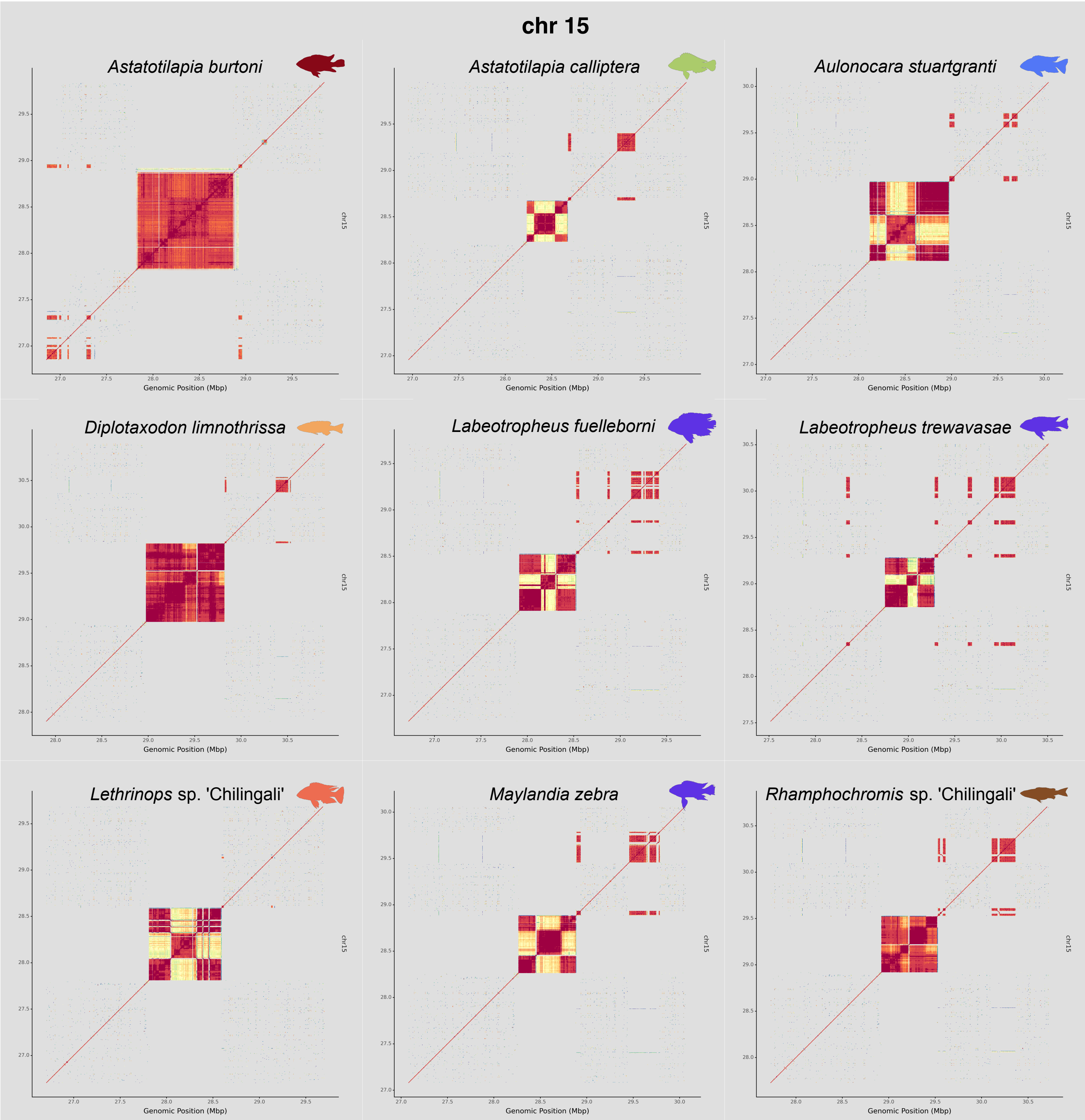

Supp. Fig. 23.- Centromeres of chr 16

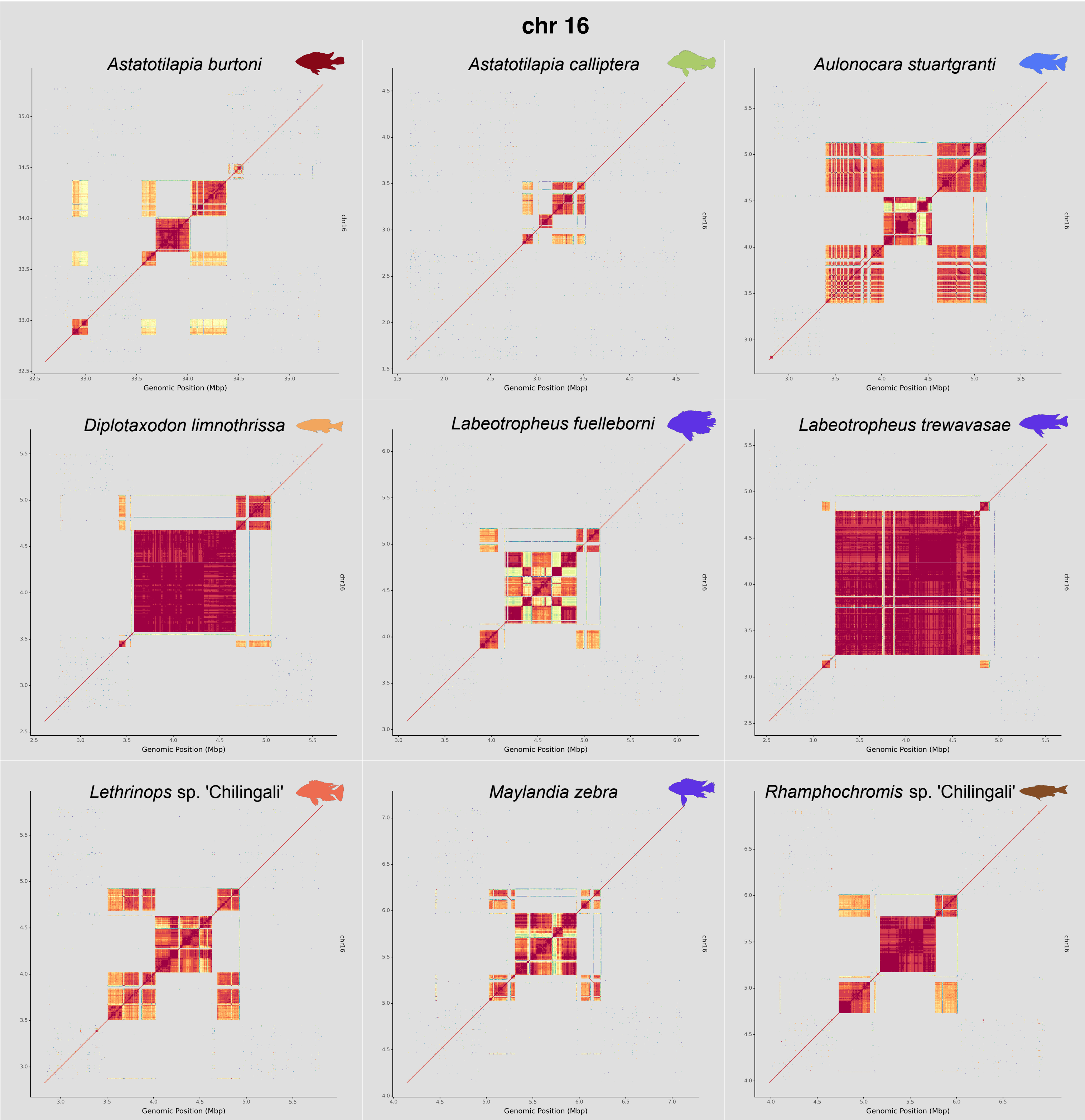

Supp. Fig. 24.- Centromeres of chr 17

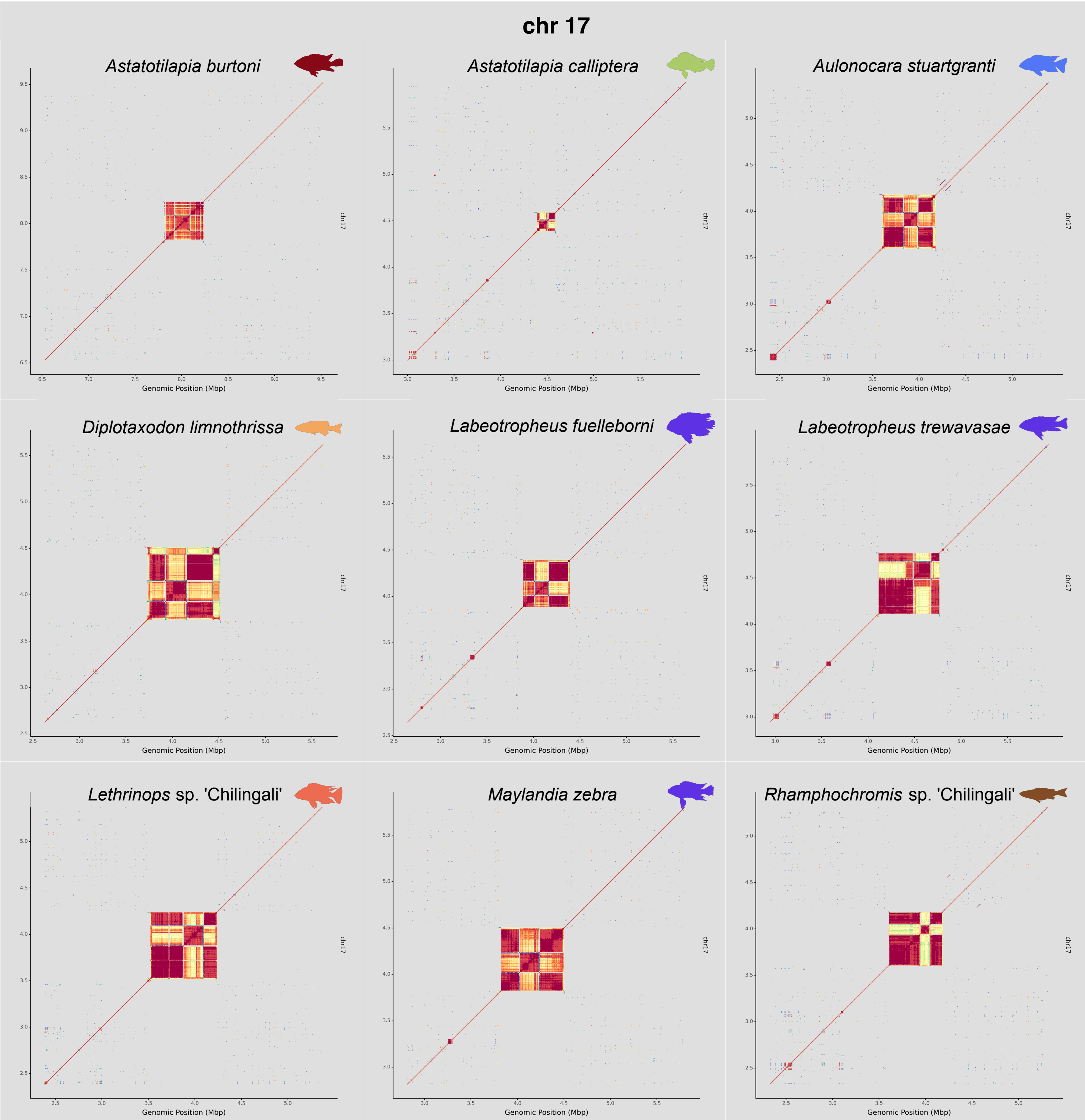

Supp. Fig. 25.- Centromeres of chr 18

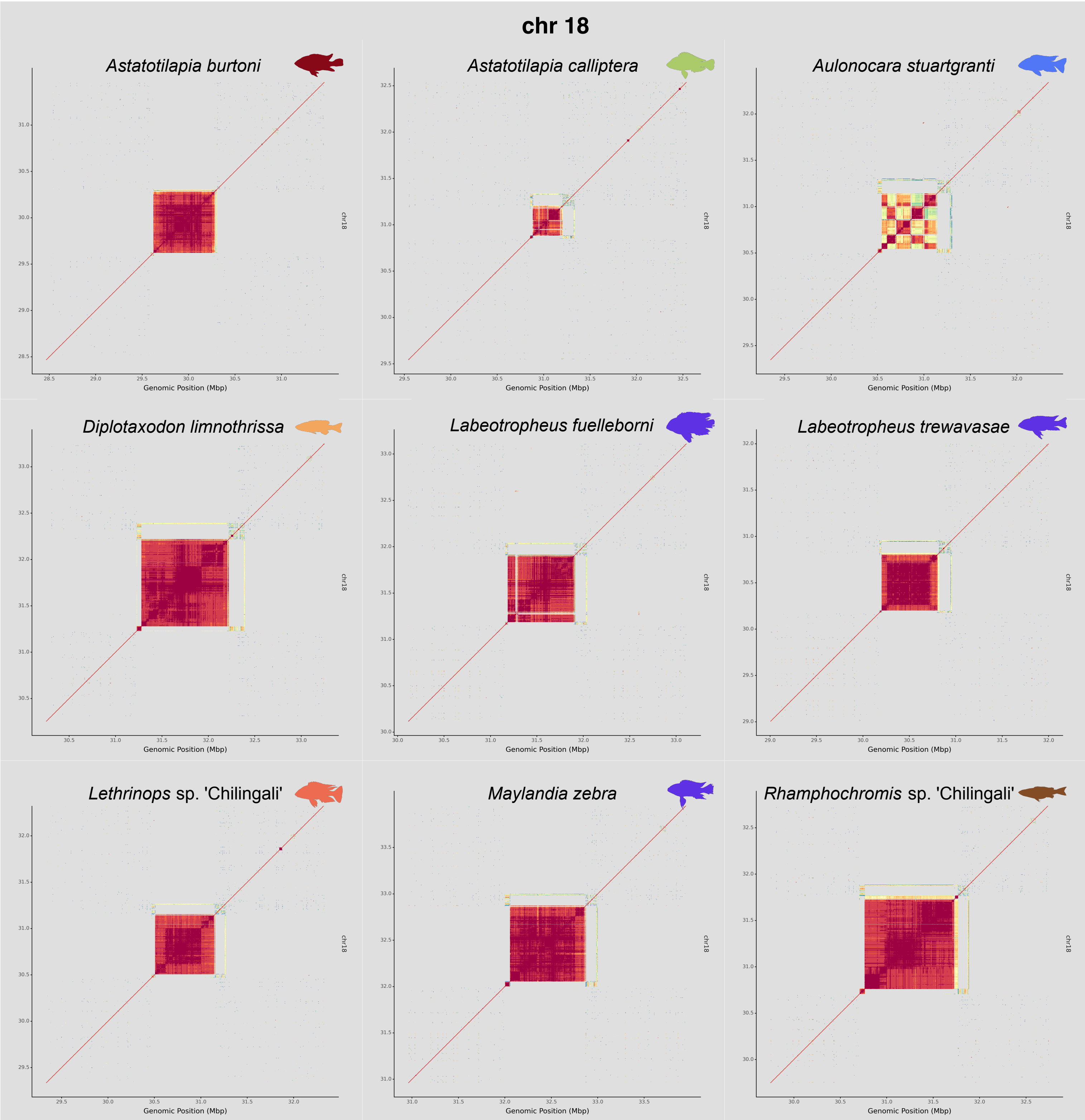

Supp. Fig. 26.- Centromeres of chr 19

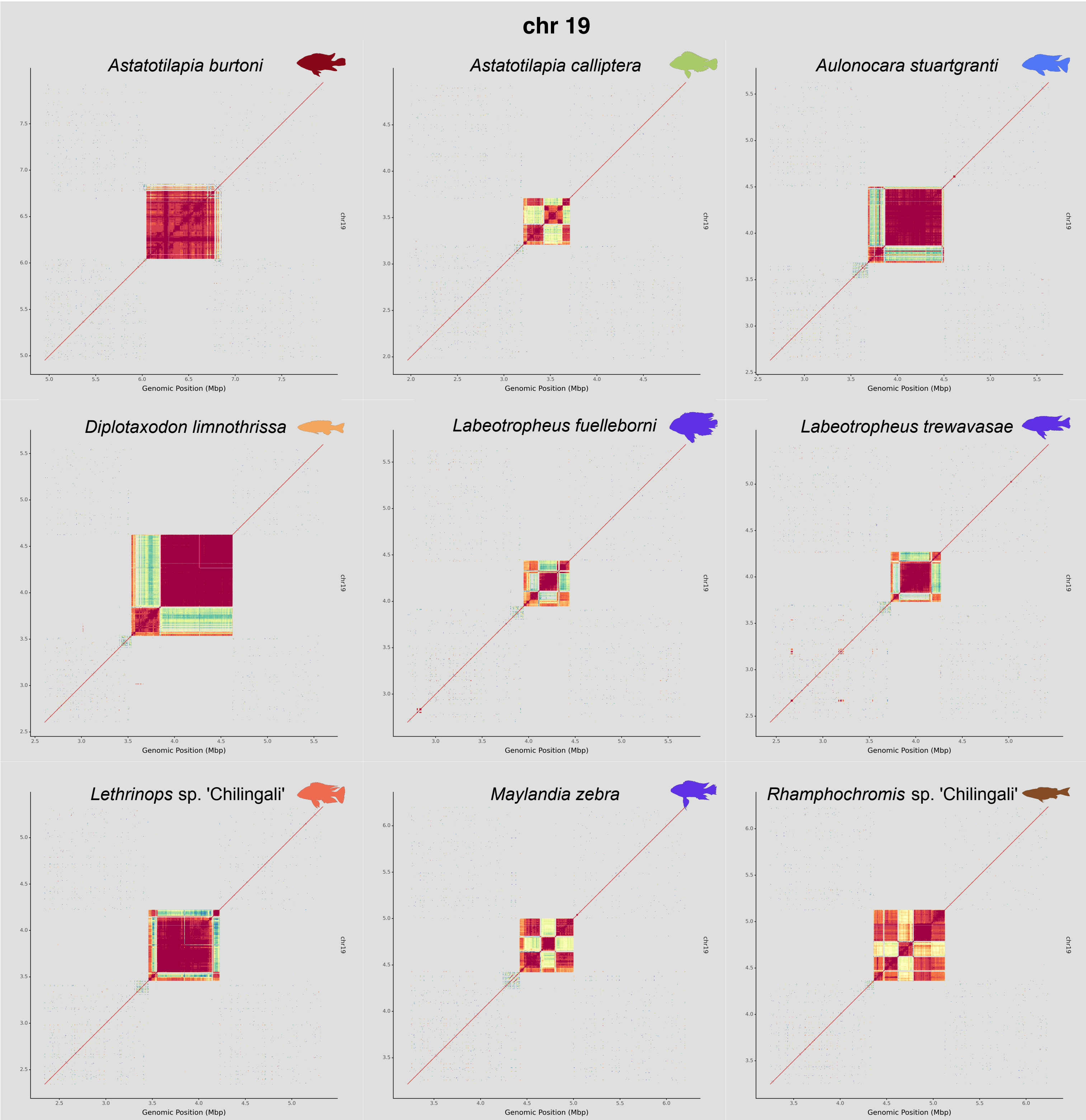

Supp. Fig. 27.- Centromeres of chr 20

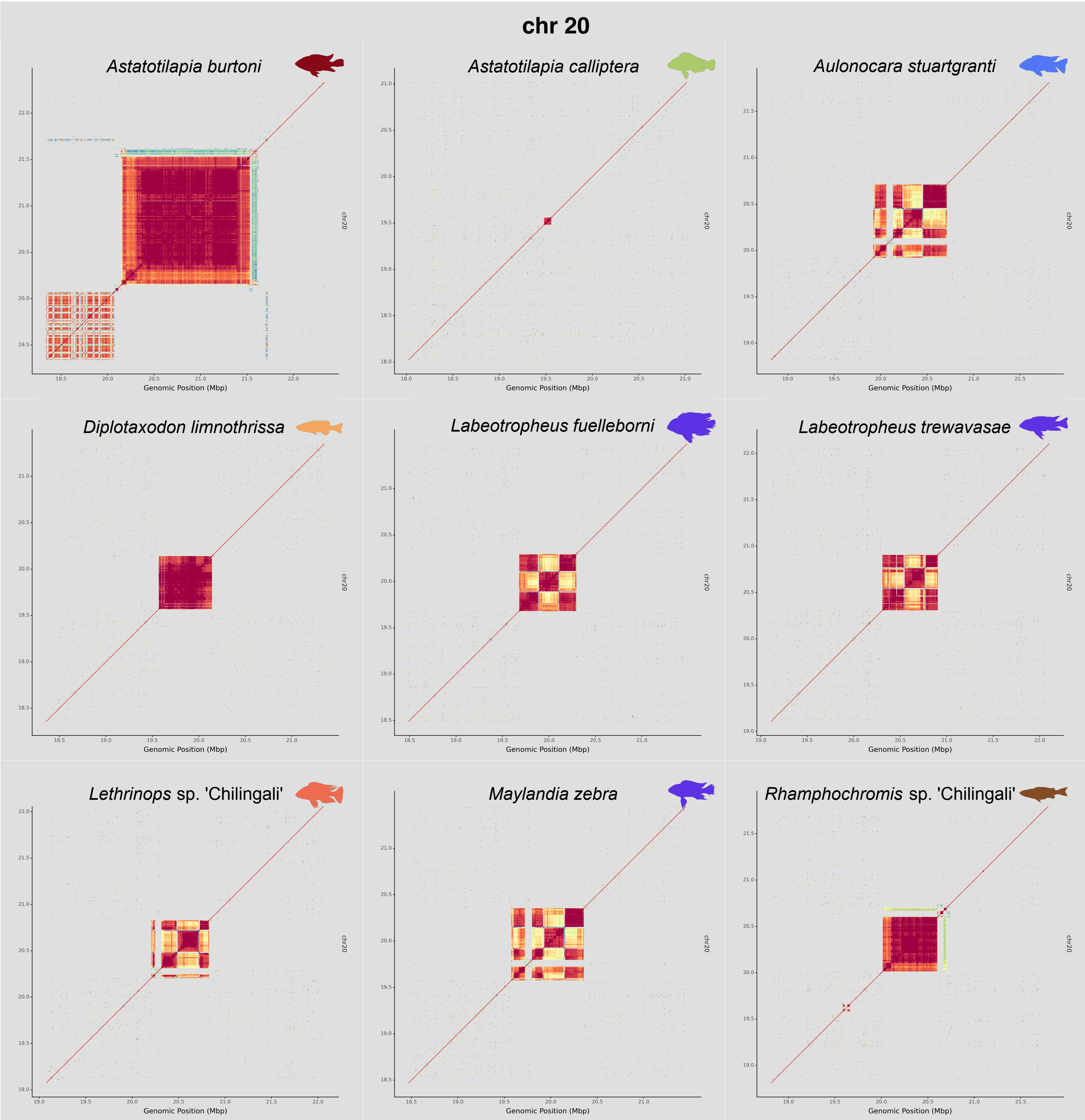

Supp. Fig. 28.- Centromeres of chr 22

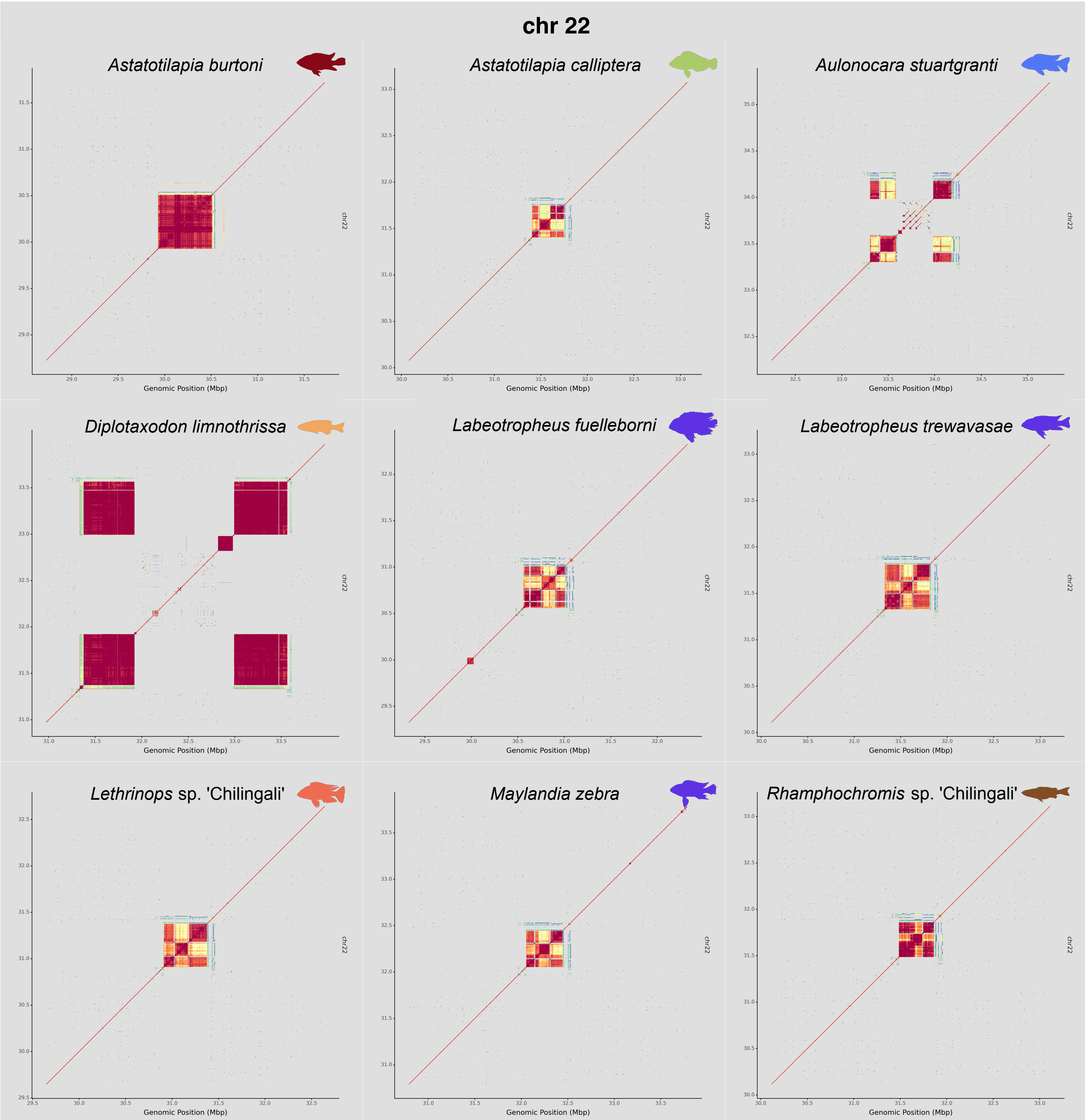

Supp. Fig. 29.- Centromeres of chr 23

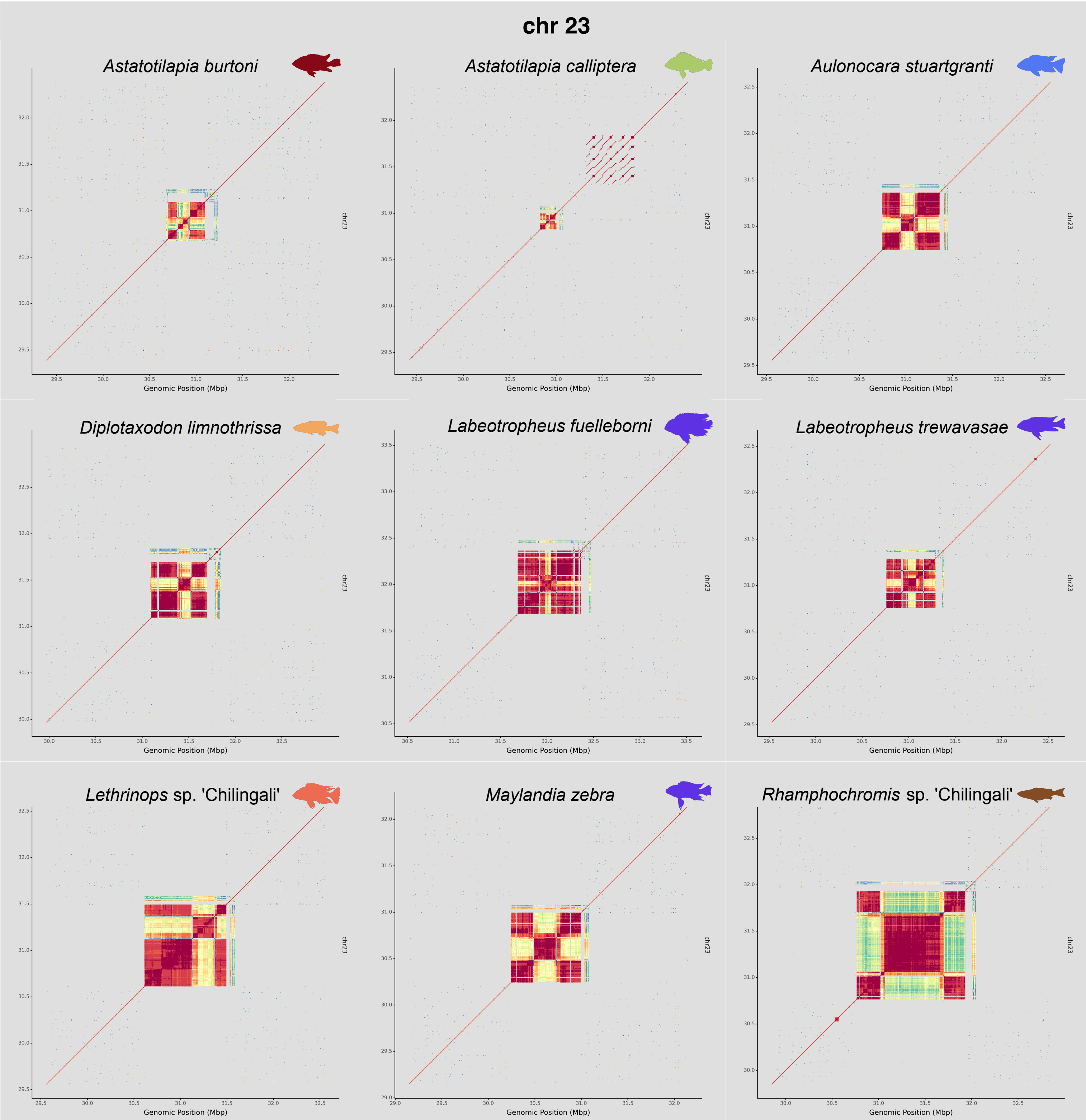

Supp. Fig. 30.- Correlation between centomere size and presence or absence of centroids, in ungapped and gapped centromeres.

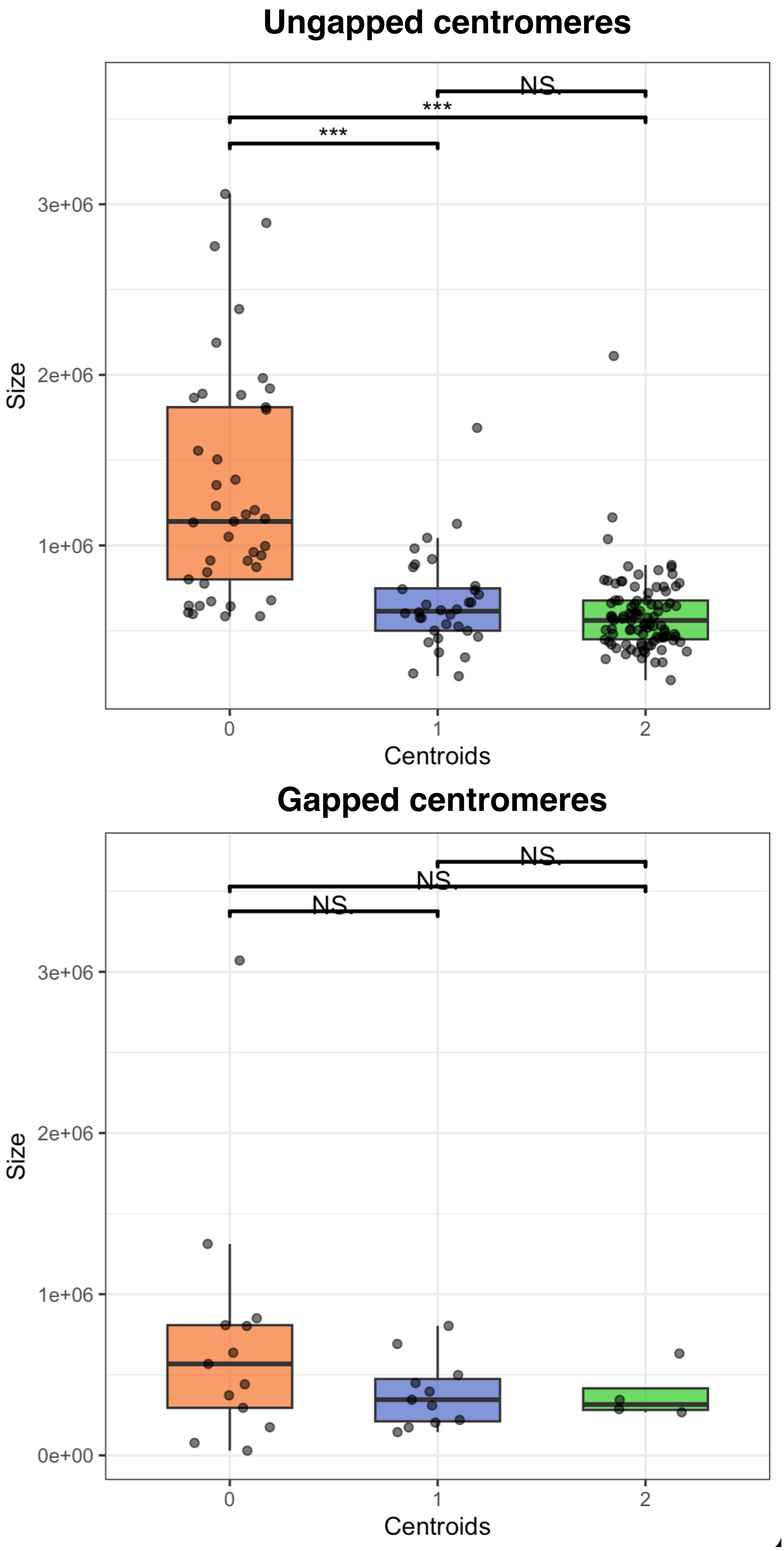

Supp. Fig. 31.- Cosegregating mutations in each of the four tandem repeats flanking centroids on chromosome 7 of *Lethrinops* sp. 'Chilingali'

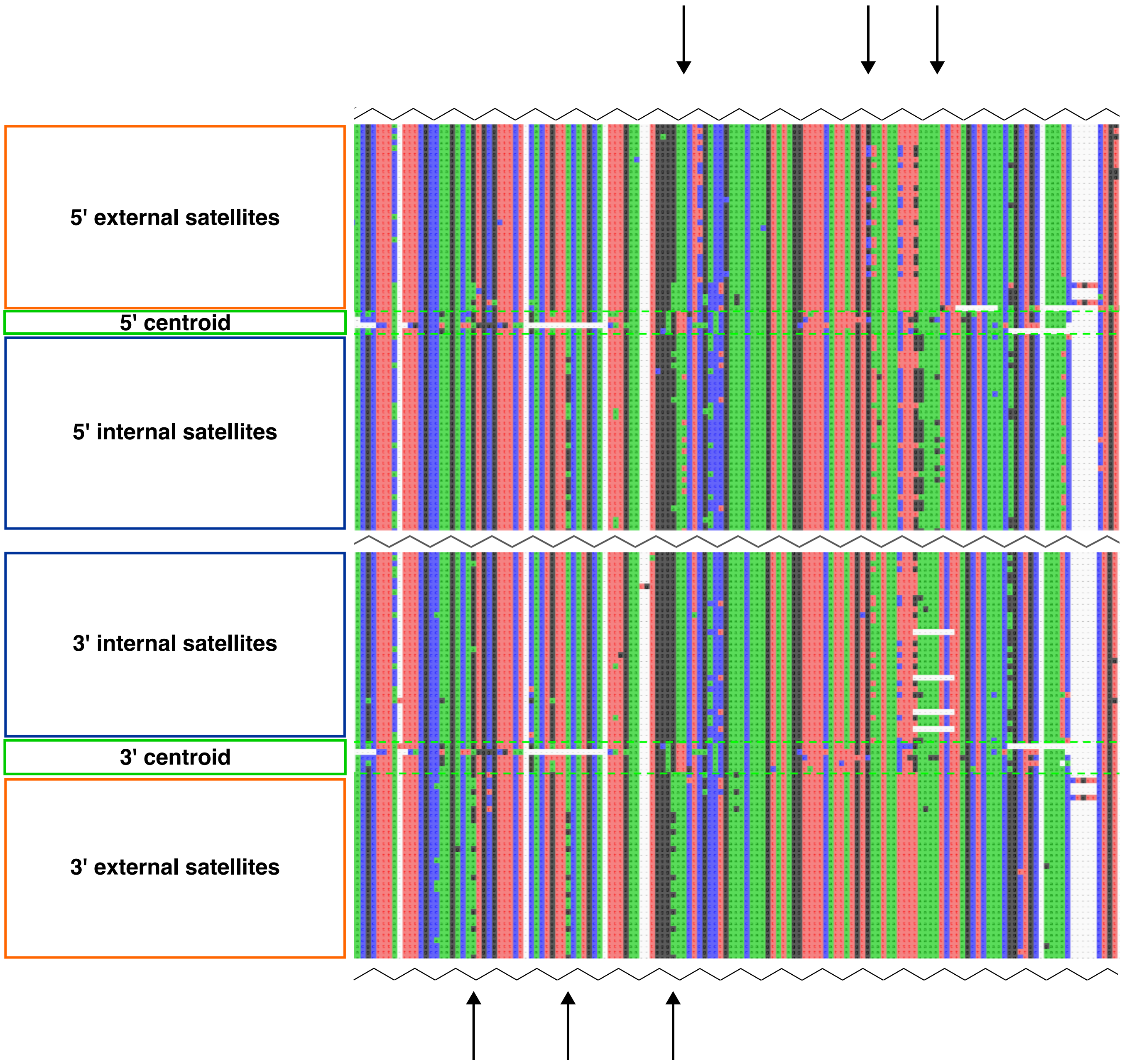

Supplement: Supplementary file 1 [file 10577_2026_9819_MOESM1_ESM.pdf]
